# Detroit, September, 1900

**Published:** 1900-01

**Authors:** 


### DETROIT, SEPTEMBER, 1900.

AFTER an earnest battle for the place of meeting in 1900, Detroit has won this distinction by a vote of 7 to 6, Minneapolis leading as second place. In the selection of Detroit the Association has decided upon a city and within the borders of a State that has never had the the value and impetus of a meeting of the Association. It will reach through Detroit a section of the United States and Canada where a large number of the profession reside and too small a number members of the Association. Detroit is a splendid convention city. It has abundant railroad facilities. It is well supplied with hotels. Her people are hospitable, and the city has charms to rouse the interest and contribute to the pleasure of every one.

The time is auspicious for a meeting in that State, for the profession is rent asunder with internal strifes for leadership. The value of her imperfect legislation is further imperiled by the dissensions over the appointments on her State Board and the scope of the work still a matter of conjecture. The Chief Veterinary Official of the State is openly charged with incompetency in the daily papers throughout that Commonwealth. The lack of support by the profession and the State has closed the doors of a school that endeavored to maintain the standard of the American Veterinary Medical Association, and sent from the State one of the ablest veterinarians she possessed. A rival school, the outcome of the high course at Detroit, established by one who was unable to maintain the school at the latter point, and which now maintains a curriculum that makes her graduates ineligible to membership in the American Veterinary Medical Association, and which is practically a one-man school. The State Association is not a unit on any of the leading questions of the day for the welfare of the profession, and the time is propitious, the hour one of need for the presence of and assistance of some strong power to aid in the solution of these trying conditions.

Let us hope the convention of the A. V. M. A. will prove the panacea for all these ills.

